# Women`s sexual function during the postpartum period: A systematic review on measurement tools

**DOI:** 10.1097/MD.0000000000038975

**Published:** 2024-07-26

**Authors:** Nazanin Rezaei, Zahra Behboodi Moghadam, Atbin Tahmasebi, Safoura Taheri, Masoumeh Namazi

**Affiliations:** a School of Nursing and Midwifery, Tehran University of Medical Sciences, Tehran, Iran; b Department of Midwifery, Ilam University of Medical Sciences, Ilam, Iran; c Department of Midwifery and Reproductive Health, School of Nursing and Midwifery, Tehran University of Medical Sciences, Tehran, Iran; d School of Medicine, Student Research Committee, Ilam University of Medical Sciences, Ilam, Iran.

**Keywords:** measurement tools, postpartum, review, sexual function, sexual health, women

## Abstract

**Background:**

Sexual health is a critical component of overall well-being, yet discussions around sexual function, especially in the context of postpartum recovery, are often taboo or sidelined. The aim was to review measurement tools assessing women’s sexual function/health during the postpartum period.

**Methods:**

We did a systematic search according to preferred reporting items for systematic reviews and meta-analyses 2020 guidelines in different databases, including PubMed, Web of Science, Scopus, Embase, ProQuest and Open Access Thesis and Dissertations, and Google scholar search engine until June 2023. Also, the reference list of the related reviews has been screened. Eligible studies included observational studies or clinical trials that evaluated women`s sexual function during the postpartum period using existing tools. Data extraction covered study characteristics, measurement tools, and their validity and reliability.

**Results:**

From 3064 retrieved records, after removing duplicates and excluding ineligible studies, and reviewing the reference list of the related reviews, 41 studies were included in this review. Tools measuring sexual function were developed from 1996 to 2017. Sexual activity questionnaire, female sexual function index (FSFI), sexual function questionnaire, short form of the pelvic organ prolapse/urinary incontinence sexual questionnaire, sexual health outcomes in women questionnaire, shorter version of FSFI, and sexual function questionnaire’s medical impact scale and Carol scale.

**Conclusion:**

Sexual activity questionnaire, FSFI, sexual function questionnaire, short form of the pelvic organ prolapse/urinary incontinence sexual questionnaire, sexual health outcomes in women questionnaire, shorter version of FSFI, sexual function questionnaire’s medical impact scale, and Carol scale are valid and reliable measuring tools to assess sexual function or sexual health during postpartum period, which can be used in primary studies according to the study aim and objectives.

## 1. Introduction

Sexuality is indeed a multifaceted aspect of human experience influenced by various factors, including mental, physical, and social dynamics.^[1]^ Throughout a woman’s life, the sense of sexual satisfaction can fluctuate, especially during significant periods such as pregnancy.^[2]^ Research indicates that issues with sexuality may arise at any stage of pregnancy, with the highest prevalence occurring in the third trimester, during the puerperium (postpartum period), and early motherhood.^[3]^ The female sexual response cycle is divided into 4 phases: desire, arousal, orgasm, and resolution. Disturbance in any of these phases results in sexual dysfunction.^[4]^ The 10th revision of the World Health Organization’s International Statistical Classification of Diseases and Related Health Problems defined sexual dysfunction as people’s inability to participate in sexual relationships as they wish.^[5]^ Female sexual dysfunction is common, affecting 40% to 45% of women. Despite the high prevalence of female sexual dysfunction worldwide, there are very limited data concerning sexual dysfunction in postpartum women and its associated risk factors.^[6]^ There are many factors, which contribute to sexual dysfunction in women. Biological changes during pregnancy and childbirth include hormonal fluctuations, physical discomforts, and alterations in genital anatomy. These physiological changes can impact sexual desire, arousal, and satisfaction. Psychological and social factors also play significant roles in shaping sexual experiences during pregnancy and postpartum.^[3]^ Women may experience emotional fluctuations, body image concerns, anxiety about childbirth, and adjustments to their new roles as mothers. Additionally, changes in social dynamics within the couple’s relationship, including shifts in intimacy, communication patterns, and division of caregiving responsibilities, can influence sexual satisfaction and overall relationship satisfaction.^[3,7–9]^

For measuring sexual function after postpartum, several measurement tools have been developed to assess women’s sexual function during the postpartum period. For instance, female sexual function index (FSFI) is a validated questionnaire consisting of 19 items covering 6 domains: desire, arousal, lubrication, orgasm, satisfaction, and pain. It has been widely used in various populations, including postpartum women.^[10]^ However, current tools vary in terms of the domains they assess, the number of items, and their sensitivity to changes in sexual function. Researchers and clinicians need to select the most appropriate tool based on the specific research questions or clinical needs. Despite the importance of tools to evaluate sexual performance, there is still a study that examines different tools to evaluate sexual performance after pregnancy. Thus, this was conducted with the aim of reviewing the women`s sexual function during the postpartum period.

## 2. Materials and methods

### 2.1. Data sources and searches

In this systematic review, we did a comprehensive search in various databases, including PubMed, Web of Science, Scopus, Embase, ProQuest, and Open Access Thesis and Dissertations, as well as Google scholar search engine. The search strategy for the PubMed database was developed as follows: (“postpartum period”[tiab] OR Postpartum[tiab] OR “Postpartum Women”[tiab] OR (Women[tiab] AND Postpartum[tiab]) OR Puerperium[tiab] OR “After delivery”[tiab] OR “After childbearing”[tiab] OR “after childbirth”[tiab]) AND (“sexual function*”[tiab] OR (Behavior[tiab] AND Sexual[tiab]) OR “Sexual Activit*”[tiab] OR (Activit*[tiab] AND Sexual[tiab]) OR “sexual dysfunction*”[tiab] OR “Orgasmic Disorder*”[tiab] OR ”Desire Disorder”[tiab] OR (Disorder*[tiab] AND Orgasmic[tiab]) OR “Sexual Arousal Disorder*”[tiab] OR (Disorder*[tiab] AND “Sexual Arousal”[tiab]) OR (“Sexual Dysfunction*”[tiab] AND Physiological[tiab]) OR “Sexual health”[tiab]). Then, we modified it for other databases as shown in Table S1, Supplemental Digital Content, http://links.lww.com/MD/N247. Also, the reference list of the related reviews has been screened.^[11,12]^

### 2.2. Study selection

Two authors (N.R., M.N.) independently reviewed the retrieved studies based on their titles and abstracts to determine their eligibility for inclusion in the study. After that, the full texts of the papers were evaluated. Observational studies or clinical trials that evaluated the sexual function/ health of women during the postpartum period using existing tools were included. Review studies, studies that used a checklist to measure postpartum sexual function, studies with language other than English, and studies with unavailable full texts were excluded from the review process. It should be noted that any disagreements were resolved by the third author (Z.B.M.) through discussion.

### 2.3. Data extraction

The data extraction form included the following variables: first author’s name, publication year, study country, study design, study population, sample size, sampling method, study outcome and its measurement tool, and outcome definition. After reviewing the primary studies that used the tools measuring sexual function, we summarized the characteristics of the measurement tools in terms of items, domains, validity, reliability, and validated versions through a separate table. Two authors extracted the data (NR, ST) from the included studies, and any disagreements were resolved by the third author (AT) through discussion.

### 2.4. Risk of bias assessment

Risk of bias assessment^[13]^ was done using the JADAD scale for reporting randomized controlled trials,^[14]^ and Newcastle–Ottawa scale^[15]^ for cohort and cross-sectional studies.^[16,17]^ Risk of bias assessment was performed by 2 independent authors (NR, ST) and a third person^[18]^ resolved any disagreements. For JADAD scale, the quality score was between 0 and 5, which was categorized into 0 to 2 as low quality, 3 as medium quality, and 4 to 5 as high quality.^[14]^ For Newcastle–Ottawa scale, Both scales have 7 items, in which 1 and zero points were given for the fulfillment of each of the 7 criteria or not, respectively. Then, the total quality score was calculated by the summation of those 7 points, and scores more than 3 was considered as high quality.^[16,17]^

### 2.5. Synthesis methods

Due to study aim, we did not do meta-analysis. Therefore, we summarized the study results, descriptively through tables.

## 3. Results

**Figure 1.**
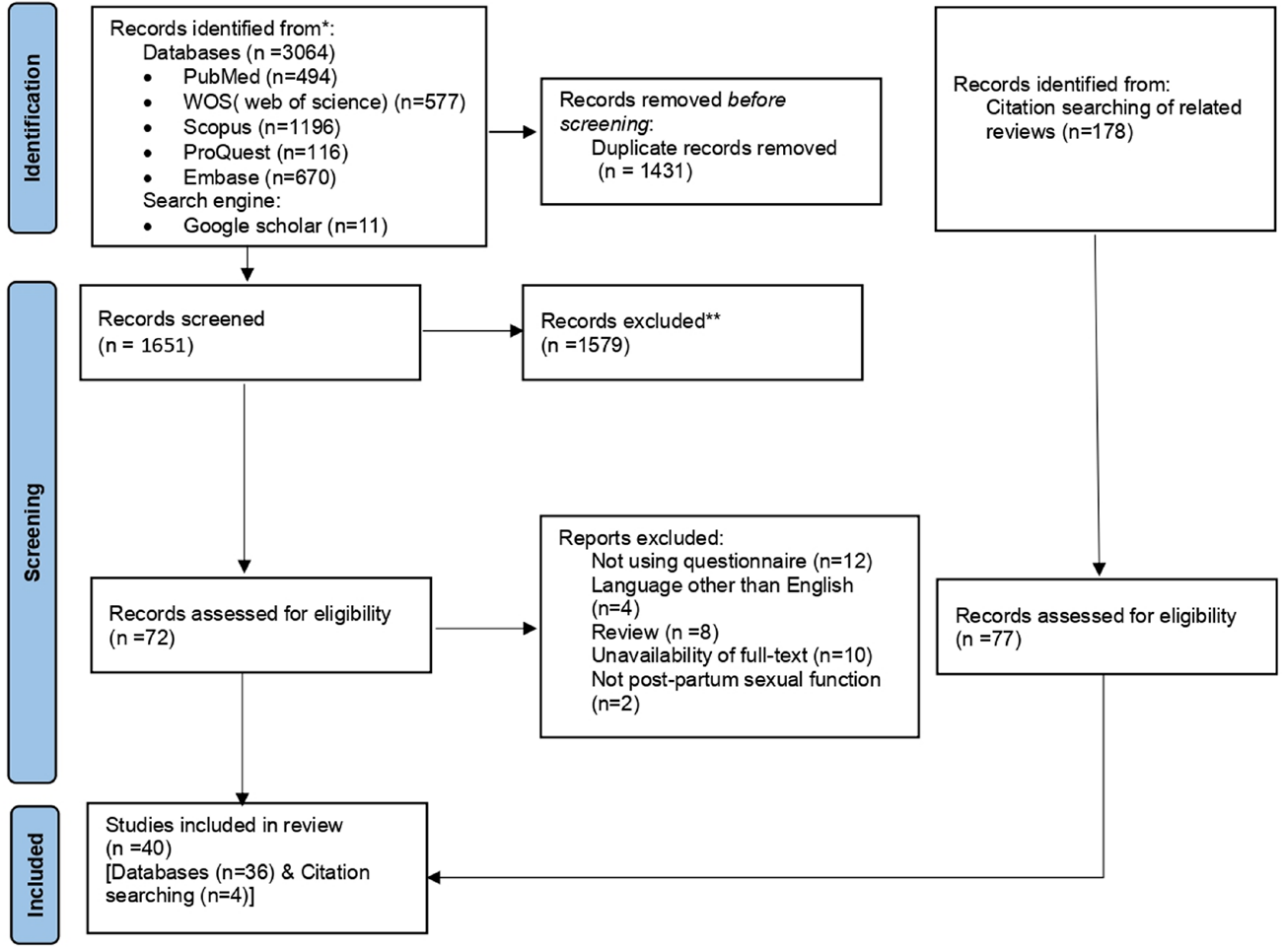
PRISMA 2020^[13]^ flow diagram of the process of searching, screening, and selecting records. PRISMA = preferred reporting items for systematic reviews and meta-analyses, WOS = Web of Science.

Figure 1 shows the flow diagram of searching, screening, and selecting process of this review. From 3064 retrieved records, after removing duplicates and excluding ineligible studies, and reviewing the reference list of the related reviews, 40 studies were included in this review. Not using questionnaire, language other than English, review studies, unavailability of full-text, and not postpartum sexual function were the reasons to exclude studies from this review.

The characteristics of the included studies according to the measuring tools are listed in Table S2, Supplemental Digital Content, http://links.lww.com/MD/N247. In addition, the items for quality assessment of included studies according to the study type (i.e., cohort, cross-sectional, and trial) were presented as Tables S3 to S5, Supplemental Digital Content, http://links.lww.com/MD/N247. Table 1 shows characteristics of the tools measuring sexual function/sexual health during postpartum period. Tools measuring the sexual function were developed from 1996 to 2017. Sexual activity questionnaire (SAQ), FSFI, sexual function questionnaire (SFQ28), short form of the pelvic organ prolapse/urinary incontinence sexual questionnaire (PISQ-12), sexual health outcomes in women questionnaire (SHOW-Q), shorter version of FSFI-6) sexual function questionnaire’s medical impact scale (SFQ-MIS), and Carol scale. The items of questionnaires varied between 5 and 28 items. In addition, they had at least 3, and at most 7 domains. The validity and reliability of the aforementioned tools were evaluated through different methods. Test-retest reliability was the most common method used to evaluate the reliability. The FSFI was the only tool that frequently used in many primary studies. It should be noted that it was the only measurement tool that had different validated versions, including Iranian, Hungarian, Taiwan, Turkish, and German version as shown in Table 1.

**Table 1:** Characteristics of the tools measuring sexual function/sexual health.

Tool	Year	Outcome	Items	Domains	Validity	Reliability	Studies used this tools	Validated versions
SAQ^[19]^	1996s	Women’s sexual functioning	9-item questionnaire	3 domains of: • Desire/pleasure from sexual intercourse • Discomfort during sexual intercourse • Habit	Good face validity and discriminating between the sexual functioning of pre- and postmenopausal women.	Reliability: test-retest reliability: 0.5 to 1.0 Pearson’s correlation coefficient: *R* = 0.65 to 1.00	Prospective cohort study^[20]^	---
FSFI^[10]^	2000	Sexual function	19-item questionnaire	5 domains of: • Desire and subjective arousal • Lubrication • Orgasm • Satisfaction • Pain/discomfort (desire: Q1–2, arousal: Q3–6, lubrication: Q7–10, orgasm: Q11–13, satisfaction: Q14–16, and pain: Q17–19).	Construct validity: good discriminant ability	Internal consistency: Cronbach’s α statistic = 0.97 Test-retest reliability: *R* = 0.88	Clinical trial^[21–23]^ Cross-sectional study^[18,24–37]^ Prospective cohort study^[20,38–49]^	Iranian version of 19-item FSFI^[18,50–52]^ Hungarian version of 19-item FSFI^[35]^ Taiwan version of 19-item FSFI^[44,53]^ The Turkish version of 19-item FSFI^[46,54]^ German version of Female Sexual Function Index (FSFI-d)^[38,55]^
SFQ28^[56]^	2002	Sexual health	28-item questionnaire	7 domains of: • Desire • Physical arousal-sensation • Physical arousal-lubrication • Enjoyment • Orgasm • Pain • Partner relationship	Excellent discriminant validity (significant difference between baseline mean SFQ domain scores of patients with sexual dysfunction compared with those without (*P* < .001)	The internal consistency: 0.65 to 0.91; test-retest reliability: 0.21 to 0.71 for Cohen’s weighted kappa and Pearson’s correlation coefficient: *R* = 0.42 to 0.78	Cross-sectional study^[57]^	----
PISQ-12^[58]^	2003	Sexual function in women with pelvic organ prolapse and/or urinary incontinence	12-item questionnaire	3 domains of: • Behavioral emotive • Physical • Partner-related	Validity: Verified by correlation with other instruments, and demographic information obtained from the patients.	Reliability: moderate to high Weighted kappa values: 0.56 to 0.93 Test-retest reliability: *R* > 0.92	Prospective cohort study^[59,60]^	----
SHOW-Q^[61]^	2008	Impact of pelvic problems on sexual function	12-item scale	4 domains of: • Satisfaction • Orgasm • Desire • Pelvic problem • Interference	Baseline correlations of SHOW-Q with health-related quality of life and symptom resolution	High internal consistency: Cronbach’s alpha = 0.86	Prospective cohort study^[62]^	----
FSFI-6^[63]^	2010	Sexual function	6-item questionnaire	5 domains of: • Desire and subjective arousal • Lubrication • Orgasm • Satisfaction • Pain/discomfort Short form included: Q2, Q4, Q7, Q11, Q16, and Q17	Discriminant ability by ROC curve	Reliability: test-retest method (*R* = 0.95)	Clinical trial^[64]^	---
SFQ-MIS^[65]^	2013	Sexual functioning changes after birth	5-item questionnaire	5 items of: • Adjustment • Impact on sex life • Interest or desire • Sexual arousal • Orgasm	Construct Validity	Cronbach’s α: 0.82 (acceptable)	Web-based survey^[66]^ Cross-sectional study^[67]^	----
Carol Scale^[68]^	2017	Postpartum sexual function and dyspareunia in women who require perineal repair after vaginal delivery	11-items questionnaires	4 factors of: • Pain/discomfort related to vaginal intercourse • Pain/discomfort on caressing the vulval area • Pain/discomfort after vaginal intercourse • Preparation for the sexual activity	Construct validity: through an exploratory factor analysis	Cronbach’s α 0.79	---	----

FSFI = female sexual function index, FSFI-6 = female sexual function index, PISQ-12 = Short form of the pelvic organ prolapse/urinary incontinence sexual questionnaire, SAQ = Sexual Activity, Questionnaire, SFQ28 = Sexual Function Questionnaire, SFQ-MIS = Sexual Function Questionnaire’s Medical Impact Scale, SHOW-Q = Sexual Health Outcomes in Women Questionnaire.

## 4. Discussion

The studies that we examined in this systematic review propose 8 different tools that were measuring sexual function/sexual health during postpartum period. Most of tool measuring sexual function and only 1 instrument evaluates sexual health after childbirth. The domains these tools evaluate, the quantity of items they provide, and how sensitive they are to variations in sexual function are all different. The best tool is frequently chosen by researchers and clinicians in accordance with the particular research objectives or clinical requirements. Furthermore, some studies might employ a variety of methods to offer a more thorough evaluation of postpartum sexual function.

Women’s sexual function in the postpartum time is important for a variety of reasons, including the individual and the family. After giving delivery, women go through a period of physical and psychological recuperation called the postpartum period. Physical changes, including hormone shifts, vaginal injuries, and weariness, can have an impact on sexual function.^[31,69]^ It is crucial to address sexual concerns during this time to support women’s general health and facilitate their healing.^[70,71]^ The postpartum sexual function of women can potentially have an effect on the mental health of mothers. Anxiety, melancholy, and low self-esteem can all be influenced by sexual dysfunction or unhappiness.^[72]^ Positive sexual experiences, on the other hand, might enhance emotions of self-worth, contentment, and general well-being.^[73]^

A comprehensive instrument for evaluating sexual function after childbirth should ideally cover multiple domains to provide a thorough assessment of women’s postpartum sexual health.^[7,74]^ One of these domains is sexual desire. The interest or motivation of a woman to participate in sexual activity is evaluated in this category. It includes both responsive and irrational sexual desire.^[75]^ Among the included tools, SAQ, FSFI, SFQ28, SHOW-Q, and FSFI-6 considered this domain in these tools. FSFI is a validated questionnaire used to assess sexual function in women across multiple domains including desire, arousal, lubrication, orgasm, satisfaction, and pain. It covers a wide range of sexual function domains, allowing for a comprehensive evaluation. It has been extensively validated and is widely used in both clinical and research settings. But, it may be relatively time-consuming to administer and score. Additionally, it may not capture all aspects of sexual function relevant to every individual. A study by Lee and Lu showed that the categories of sexual dysfunction that most commonly experienced in postpartum women was lack of sexual desire.^[76]^ Heidari et al determined that mothers experience a decline in sexual desire after delivery. So, in fact, sexual desire is a crucial component of postpartum sexual health, and evaluating it can give important insights into what it is like for women to adjust to life after childbirth.^[77]^ FSFI-6 is a shorter version of the FSFI questionnaire, focusing on 6 key domains of sexual function: desire, arousal, lubrication, orgasm, satisfaction, and pain. It offers a more streamlined assessment compared to the full FSFI, making it quicker and easier to administer. Thin questionnaire may sacrifice some level of detail and comprehensiveness compared to the full FSFI, potentially missing important nuances in sexual function.^[63]^ Another scale that covered the desire is the Carol Scale, also known as the Carol Scales of Sexuality, is a tool designed to measure various dimensions of sexuality, including sexual desire, arousal, and satisfaction. It offers a multidimensional assessment of sexuality, encompassing both physical and psychological aspects^.[68]^

The physiological and psychological reactions that prime the body for sexual activity are referred to as sexual arousal. It encompasses both bodily reactions like lubrication and genital stimulation as well as subjective experiences of sexual desire.^[78]^ Among the included tools, FSFI, SFQ28, FSFI-6, and SFQ-MIS assessed sexual arousal in their measurement. Other studies have highlighted desire, arousal, and orgasm as the most common sexual problems encountered during the postpartum period.^[8,79]^ In Khajehei’s study of 325 Australian women who had given birth within 1 year, it was reported that sexual desire disorder was the most common (81.2%) sexual problem, followed by orgasmic problems (53.5%) and sexual arousal disorder (52.3%).^[80]^ For sexual action to be pleasant and enjoyable, lubrication is necessary. Evaluating a woman’s capacity to generate natural lubrication or her requirement for synthetic lubricants is part of the assessment process for this domain. Furthermore, Orgasm Function evaluates a woman’s capacity to experience orgasm during intercourse. It covers the number and strength of orgasms as well as any obstacles or lag times encountered in reaching an orgasm.^[62,81]^ SFQ28, FSFI, and FSFI-6 assessed lubricant domain and most of the included tools considered Orgasm function as 1 part of their assessment. According to recent research, many women have changed sexual health following the delivery of their first child. These changes can include dyspareunia, decreased interest in sexual engagement, and loss of vaginal lubrication.^[82,83]^ A review study suggests that there is a decline in sexual function following childbirth compared to prepregnancy. In particular, the studies show an increase in perineal pain and dyspareunia, a reduction in sexual desire and in the capacity for sexual arousal, and less intensity and duration of orgasm in the first 3 months postpartum, with a gradual improvement in the 6 months postpartum.^[84–86]^ The FSFI is a widely used questionnaire designed to assess various domains of sexual function in women. One of the key domains it evaluates is arousal. Arousal is one of the domains covered by the FSFI questionnaire. It typically includes questions related to both subjective arousal (how sexually aroused the individual feels) and physiological arousal (physical changes associated with arousal, such as lubrication). The arousal domain of FSFI may consist of questions about the frequency of arousal, the ease of becoming sexually aroused, and the level of arousal experienced during sexual activity. FSFI provides a comprehensive evaluation of arousal by examining both subjective and physiological aspects. This allows for a more holistic understanding of a woman’s arousal experiences.^[9]^

The other important domains of sexual function that are very important and should consider as vital key for defection of sexual disorder are sexual satisfaction and sexual pain. Sexual satisfaction reflects a woman’s subjective evaluation of her sexual experiences and overall fulfillment with her sexual relationship. It encompasses emotional and physical aspects of sexual pleasure and connection. Sexual pain evaluates the presence and severity of any pain or discomfort experienced during sexual activity. This may include pain related to vaginal dryness, scar tissue from childbirth, or other physical conditions.^[9,87]^ Parents frequently mentioned issues with arousal, desire, and sexual satisfaction as well as a decline in relationship satisfaction. One of the most prevalent sexual dysfunctions in the postpartum phase is libido loss.^[88,89]^ The degree of sexual satisfaction was also found to be impacted by low sex drive. The initial postpartum visit should ideally occur sooner than 6 weeks after the woman has given birth.^[89]^ SAQ is a questionnaire designed to assess various aspects of sexual activity, including frequency, satisfaction, and types of sexual behaviors. It provides a comprehensive overview of sexual activity patterns and behaviors. It can be useful for both clinical assessment and research purposes. However, it relies heavily on self-reporting, which can be subject to biases such as social desirability bias or recall bias. Additionally, it may not capture nuanced aspects of sexual function or satisfaction.^[19]^

In addition, the reduction in sexual satisfaction, both emotionally and physically, and reduction in sexual variability postpartum suggests that providers should include some conversation about sexual satisfaction in the early postpartum period. In particular, women experiencing postpartum dyspareunia (difficult sex) frequently worry about sexual pain. Promoting postpartum sexual health requires addressing the various causes that lead to sexual pain following childbirth. The process of childbirth, especially vaginal delivery, can cause trauma to the perineum, vaginal tissues, and pelvic floor muscles. This trauma may result in pain or discomfort during sexual activity in the postpartum period. Episiotomy or tears during delivery can exacerbate this pain. Hormonal changes can cause vaginal dryness and decreased lubrication, which can make sexual activity uncomfortable or painful. One such hormonal change is a drop in estrogen levels following childbirth.^[62]^ Additionally, estrogen levels, which are influenced by breastfeeding, may also have an impact on vaginal dryness and pain during sexual activity. Sexual desire, arousal, and arousal can be affected by the emotional and psychological transitions of being a new mother, such as stress, exhaustion, worry, and changes in body image. These changes can also exacerbate sexual pain.^[1]^ In this area, SFQ28 is a questionnaire designed to assess sexual function and satisfaction in individuals with chronic diseases, particularly rheumatic diseases. It addresses the specific needs of individuals with chronic diseases, providing insights into how these conditions impact sexual function. It may have limited applicability outside of the population with chronic diseases it was designed for. Additionally, it may not cover all aspects of sexual function relevant to other populations. SFQ28 typically includes questions related to satisfaction with various aspects of sexual function and experience. These may encompass satisfaction with sexual desire, arousal, lubrication (if applicable), orgasm, overall sexual function, and satisfaction with the sexual relationship. Participants are often asked to rate their level of satisfaction on a scale or provide qualitative responses.^[56]^ Among these included questionnaire, PISQ-12 is a short questionnaire specifically designed to assess sexual function in women with pelvic organ prolapse or urinary incontinence. The PISQ-12 is a shortened version of the original PISQ, designed to assess sexual function in women with pelvic floor disorders. It has undergone validation studies and demonstrated good reliability and validity in this specific population. It focuses on a specific population with unique sexual health concerns, allowing for targeted assessment and intervention.^[58]^

Generally, Among the scales you mentioned, the FSFI and PISQ-12 are commonly used and well-validated measures for assessing women’s sexual function and health during the postpartum period. The FSFI covers multiple domains of sexual function, including desire, arousal, lubrication, orgasm, satisfaction, and pain. This comprehensive approach allows for a thorough evaluation of sexual health during the postpartum period, which may involve changes in various aspects of sexual function. The PISQ-12 specifically targets sexual function in women with pelvic floor disorders, such as pelvic organ prolapses and urinary incontinence. These conditions are common during the postpartum period due to factors like childbirth-related trauma and hormonal changes. Therefore, the PISQ-12 may be particularly relevant for assessing sexual health in this population.

This study has some strengths and limitations. The results of this review enable scholars to compile and synthesize the body of knowledge already written about sexual function tools. Here, a review of the instruments used for sexual function during the postpartum period aids in consolidating knowledge about the instruments that are accessible, as well as their advantages, disadvantages, and potential areas for development. Also, helps identify gaps and limitations in the assessment of women’s sexual function after childbirth. By understanding the strengths and weaknesses of current instruments, researchers can propose enhancements or develop new tools that better capture the complexities of postpartum sexual health. The limitation of this study was that included studies’ quality can differ greatly from one another. Certain studies may have biases, small sample sizes, or methodological flaws that compromise the validity of their conclusions. Measurement tools for assessing women’s sexual function during the postpartum period may vary in terms of their design, reliability, validity, and cultural appropriateness. Comparing and synthesizing findings across different tools can be challenging due to this heterogeneity.

Overall, this study provides insight into different tolls in evaluating women’s sexual function following delivery. By assisting health care providers in making well-informed choices regarding the choice and application of suitable evaluation instruments, they eventually improve the assistance and treatment provided to new mothers.

## 5. Conclusion

SAQ, FSFI, SFQ28, PISQ-12, SHOW-Q, FSFI-6, SFQ-MIS, and Carol scale are valid and reliable measuring tools to assess sexual function or sexual health during postpartum period, which can be used in primary studies according to the study aim and objectives. Depending on the unique research objectives and population being studied, researchers can select the best method or combination of tools to assess sexual function or sexual health during the postpartum period. When choosing a measurement tool, consider issues such as population demographics, cultural considerations, and the specific components of sexual function under investigation. Furthermore, researchers should confirm that the chosen tool has been verified and shown reliable in similar groups and circumstances.

## Author contributions

**Conceptualization:** Nazanin Rezaei

**Writing—original draft:** Nazanin Rezaei, Masoumeh Namazi

**Supervision:** Zahra Behboodi Moghadam

**Data curation:** Atbin Tahmasebi, Safoura Taheri

**Formal analysis:** Atbin Tahmasebi, Safoura Taheri

**Methodology:** Safoura Taheri

**Writing—review and editing:** Masoumeh Namazi

## Supplementary Material
