# Supplementary material for: Women`s sexual function during the postpartum period: A systematic review on measurement tools
Source: Medicine (Baltimore). 2024 Jul 26;103(30):e38975. doi: 10.1097/MD.0000000000038975 (PMC11272350; doi:10.1097/MD.0000000000038975)
Supplement: Supplementary file 1 [file medi-103-e38975-s001.docx]

| Search SYNTAX in **PubMed** | | | **No. of results** | |
| --- | --- | --- | --- | --- |
| 1 | (“postpartum period”[tiab] OR Postpartum[tiab] OR “Postpartum Women”[tiab] OR (Women[tiab] AND Postpartum[tiab]) OR Puerperium[tiab] OR “After delivery”[tiab] OR “After childbearing”[tiab] OR “after childbirth”[tiab]) AND (“sexual function*”[tiab] OR (Behavior[tiab] AND Sexual[tiab]) OR “Sexual Activit*”[tiab] OR (Activit*[tiab] AND Sexual[tiab]) OR “sexual dysfunction*”[tiab] OR “Orgasmic Disorder*”[tiab] OR ”Desire Disorder”[tiab] OR (Disorder*[tiab] AND Orgasmic[tiab]) OR “Sexual Arousal Disorder*”[tiab] OR (Disorder*[tiab] AND “Sexual Arousal”[tiab]) OR (“Sexual Dysfunction*”[tiab] AND Physiological[tiab]) OR “Sexual health”[tiab]) | | 494 | |
| Search Syntax in **Web of Science** | | | | |
| 2 | TS=(“postpartum period” OR Postpartum OR “Postpartum Women” OR (Women AND Postpartum) OR Puerperium OR “After delivery” OR “After childbearing” OR “after childbirth”) AND TS=(“sexual function*” OR (Behavior AND Sexual) OR “Sexual Activit*” OR (Activit* AND Sexual) OR “sexual dysfunction*” OR “Orgasmic Disorder*” OR ”Desire Disorder” OR (Disorder* AND Orgasmic) OR “Sexual Arousal Disorder*” OR (Disorder* AND “Sexual Arousal”) OR (“Sexual Dysfunction*” AND Physiological) OR “Sexual health”) | | 577 | |
| Search Syntax in **Scopus** | | | | |
| 3 | TITLE-ABS-KEY(“postpartum period” OR Postpartum OR “Postpartum Women” OR (Women AND Postpartum) OR Puerperium OR “After delivery” OR “After childbearing” OR “after childbirth”) AND TITLE-ABS-KEY(“sexual function*” OR (Behavior AND Sexual) OR “Sexual Activit*” OR (Activit* AND Sexual) OR “sexual dysfunction*” OR “Orgasmic Disorder*” OR ”Desire Disorder” OR (Disorder* AND Orgasmic) OR “Sexual Arousal Disorder*” OR (Disorder* AND “Sexual Arousal”) OR (“Sexual Dysfunction*” AND Physiological) OR “Sexual health”) | | 1196 | |
| Search Syntax in **ProQuest** | | | | |
| 4 | | TI,AB,SU(“postpartum period” OR Postpartum OR “Postpartum Women” OR (Women AND Postpartum) OR Puerperium OR “After delivery” OR “After childbearing” OR “after childbirth”) AND TI,AB,SU(“sexual function*” OR (Behavior AND Sexual) OR “Sexual Activit*” OR (Activit* AND Sexual) OR “sexual dysfunction*” OR “Orgasmic Disorder*” OR ”Desire Disorder” OR (Disorder* AND Orgasmic) OR “Sexual Arousal Disorder*” OR (Disorder* AND “Sexual Arousal”) OR (“Sexual Dysfunction*” AND Physiological) OR “Sexual health”) | | 116 |
| Search Syntax in **Embase** | | | | |
| 5 | | (“postpartum period”:ti,ab OR Postpartum:ti,ab OR “Postpartum Women”:ti,ab OR (Women:ti,ab AND Postpartum:ti,ab) OR Puerperium:ti,ab OR “After delivery”:ti,ab OR “After childbearing”:ti,ab OR “after childbirth”:ti,ab) AND (“sexual function*”:ti,ab OR (Behavior:ti,ab AND Sexual:ti,ab) OR “Sexual Activit*”:ti,ab OR (Activit*:ti,ab AND Sexual:ti,ab) OR “sexual dysfunction*”:ti,ab OR “Orgasmic Disorder*”:ti,ab OR ”Desire Disorder”:ti,ab OR (Disorder*:ti,ab AND Orgasmic:ti,ab) OR “Sexual Arousal Disorder*”:ti,ab OR (Disorder*:ti,ab AND “Sexual Arousal”:ti,ab) OR (“Sexual Dysfunction*”:ti,ab AND Physiological:ti,ab) OR “Sexual health”:ti,ab) | | 670 |
| Search Syntax in **Google Scholar search engine** | | | | |
|  | | (“postpartum period” OR Postpartum OR “Postpartum Women” OR (Women AND Postpartum) OR Puerperium OR “After delivery” OR “After childbearing” OR “after childbirth”) AND (“sexual function*” OR (Behavior AND Sexual) OR “Sexual Activit*” OR (Activit* AND Sexual) OR “sexual dysfunction*” OR “Orgasmic Disorder*” OR ”Desire Disorder” OR (Disorder* AND Orgasmic) OR “Sexual Arousal Disorder*” OR (Disorder* AND “Sexual Arousal”) OR (“Sexual Dysfunction*” AND Physiological) OR “Sexual health”) | | 11 |
| Total | | | | 3064 |

**Table S1.** Search strategy used to retrieve related studies

| **Author (year)** | **Country** | **Study design** | **Study**  **Population** | **Sample Size** | **Outcome**  **(Definition)** | **Quality** |
| --- | --- | --- | --- | --- | --- | --- |
| **Tool: Sexual Activity Questionnaire (SAQ) ^[19]^** | | | | | |  |
| Spaich et al. (2020) ^[20]^ | Germany | Prospective cohort study | Pregnant women presenting for delivery at the University Medical Centre Mannheim | 522 women | Women’s sexual function within 12 months postpartum | High |
| **Tool: The Female Sexual Function Index (FSFI) ^[21]^** | | | | | |  |
| Smith et al. (2022) ^[22]^ | USA | Randomized controlled trial | Primiparous women with a second-degree or greater perineal laceration following a term vaginal delivery | 59 women | Sexual function at 12 weeks postpartum | High |
| Azarkish et al. (2022) ^[26]^ | Iran | Cross-sectional study | Woem living in Zanjan, vaginal birth, minimum gestational age of 37 weeks, and stable life with spouse | 216 women | Sexual function, 4 to 5 months postpartum | High |
| Surucu et al. (2022) ^[41]^ | Turkey | Prospective cohort study | Pregnant women had a normal pregnancy, did not have any sexual restrictions, did not have conditions such as placenta previa, premature rupture of membrane, or threat of premature birth | 113 women | Sexual function at 6 month postpartum | High |
| Szollosi et al. (2022) ^[50]^ | Hungary | Prospective cohort study | Women aged 18 to 45 years, in the same relationship since at least 6 months before their last pregnancy | 602 women | Sexual dysfunction during the first year postpartum | High |
| Bartels et al. (2021) ^[30]^ | USA | Survey | Women experienced a pregnancy complicated by placenta accreta spectrum, age oever 18 years, fluency in English | 142 women | Sexual function at 24-36 months postpartum compared to 0-6 month postpartum | High |
| Zgliczynska et al. (2021) ^[34]^ | Poland | Survey | Women between 10 weeks and 1 year after delivery, vaginal intercourses before pregnancy and the resumption of vaginal intercourses after delivery. | 433 women | Sexual function between 2 to 12 months after delivery | High |
| **Author (year)** | **Country** | **Study design** | **Study**  **Population** | **Sample Size** | **Outcome**  **(Definition)** | **Quality** |
| Mirzaei et al. (2021) ^[18]^ | Iran | Cross-sectional study | Iranian Pregnant, lactating, non-pregnant/non-lactating women | 603 women | Sexual function at lactating period | High |
| De Sousa et al. (2021) ^[49]^ | Portugal | Prospective cohort study | Women who had a singleton term vaginal delivery | 304 women | Sexual function at 3, 6 months and 1 year postpartum | High |
| Sheikhi et al. (2020) ^[24]^ | Iran | Clinical trial | Women aged 20 to 35 years, primiparity, healthy perineum or low‑grade tears, healthy term singleton neonate,) lack of problems before childbirth, living with the spouse at the time of study, no medical complications during the prenatal and postnatal periods | 100 women | Sexual function at 8 weeks after the intervention | Low |
| Ugwu et al. (2020) ^[42]^ | Nigeria | Prospective cohort study | All parturients who had had an uncomplicated cesarean section or a vaginal delivery at the two study centers | 91 women | Sexual function at 6 weeks and 3 months postpartum | High |
| Spaich et al. (2020) ^[20]^ | Germany | Prospective cohort study | Pregnant women presenting for delivery at the University Medical Centre Mannheim | 522 women | Women’s sexual function within 12 months postpartum | High |
| Matthies et al. (2019) ^[39]^ | Germany | Prospective cohort study | Women aged 18 years or older and having a sufficient knowledge of the German language | 150 women | Sexual function at 4 months postpartum | High |
| Szollosi et al. (2019) ^[36]^ | Hungry | Cross-sectional study | Women with singleton pregnancies, aged 18 to 45 years, currently in a relationship with their partner since at least 6 months before their latest pregnancy, and already returned to sexual life | 300 women | Sexual function at 3 months postpartum | Low |
| Szollosi et al. (2019) ^[33]^ | Hungry | Cross-sectional study | Women with singleton pregnancies, age 18–45 years, in a relationship with their partner currently and for at least 6 months before the pregnancy | 253 women | Sexual function at 3 months postpartum | Low |
| Moghadam et al. (2019) ^[53]^ | Iran | Prospective cohort study | All nulliparous mothers aged 18 to 35 years | 107 women | Sexual Function at 6 and 12 months after delivery | High |
| **Author (year)** | **Country** | **Study design** | **Study**  **Population** | **Sample Size** | **Outcome**  **(Definition)** | **Quality** |
| Banaei et al. (2018) ^[28]^ | Iran | Cross-sectional study | Lactating women who had no history of complications during pregnancy, postpartum delivery | 432 women | Sexual function between 2 to 12 months postpartum | High |
| Rezaei et al. (2017) ^[32]^ | Iran | Cross-sectional study | More than eight weeks and less than eight months post-delivery; age ≥ 18 years; delivery at week 38 to 42; couples with no history of surgery in the past 3 months and lesions or other genital injury | 380 women | Sexual function between 3 to 12 months postpartum | High |
| Kahramanoglu et al. (2017) ^[47]^ | Turkey | Prospective cohort study | Nulliparous women aged 18 to 45 years in stable relationships | 452 women | Sexual function at 3 and 6 months postpartum | High |
| Barbara et al. (2016) ^[29]^ | Italy | Cross-sectional study | Caucasian primiparous women aged 18 to 45 years old, had a body mass index lower than 30, and delivered at 37 weeks of gestation or later | 262 women | Sexual Function at 6 months after childbirth | High |
| Yıldız et al. (2015) ^[40]^ | Turkey | Prospective cohort study | Healthy pregnant women in their eighth weeks of pregnancy, planning to continue their pregnancy follow-up and delivery care up to 6 month postpartum | 59 women | Sexual Function at 6 months after childbirth | High |
| Anbaran et al. (2015) ^[31]^ | Iran | Cross-sectional study | Women aged 18-45 years, parity 5 or less, no perineal tear (grades 3 and 4) in the recent childbirth, singleton pregnancy and a healthy baby | 366 women | Sexual function at four months after childbirth | High |
| Chang et al. (2015) ^[45]^ | Taiwan | Prospective cohort study | Women aged at least 18 years with the ability to read traditional Chinese | 351 women | Sexual function at 4 to 6 weeks and at 3, 6, and 12 months after delivery | High |
| De Souza et al. (2015) ^[46]^ | Australia | Prospective cohort study | Primigravid women | 440 women | Sexual Function at 6 and 12 months postpartum | High |
| Dabiri et al. (2014) ^[25]^ | Iran | Cross-sectional study | Primiparous women in postpartum period, | 150 women | Sexual function within 3 and 6 months postpartum | Low |
| **Author (year)** | **Country** | **Study design** | **Study**  **Population** | **Sample Size** | **Outcome**  **(Definition)** | **Quality** |
| Lurie et al. (2013) ^[48]^ | Israel | Prospective cohort study | Healthy, postpartum women aged 18 to 45 | 82 women | Sexual function at 6, 12, and 24 weeks postpartum | High |
| Baghdari et al. (2012) ^[27]^ | Iran | Cross-sectional study | Age 18 to 45 years and less than 5 pregnancy | 366 women | Sexual function at 4 months postpartum | Low |
| Hosseini et al. (2012) ^[37]^ | Iran | Cross-sectional study | Healthy women with antenatally normal singleton pregnancies at term underwent normal vaginal delivery or planned cesarean section | 213 women | Sexual function at 6 to 24 months postpartum | High |
| Citak et al. (2010) ^[23]^ | Turkey | Randomized trial | Primiparous women | 75 women | Sexual function in the 4th and 7th postpartum month | High |
| Shirvani et al. (2010) ^[35]^ | Iran | Cross-sectional study | Married and literate women | 490 women | Sexual function at one year postpartum (3 to 12 months from child birth) | Low |
| Klein et al. (2009) ^[38]^ | Austria | Cross-sectional study | Primiparae with liveborn singletons at term (≥37 weeks of gestation) and cephalic presentation who had delivered vaginally or who underwent elective cesarean section | 99 women | Sexual function at 12 to 18 months after childbirth | High |
| Baksu et al. (2007) ^[43]^ | Turkey | Prospective cohort study | Primiparous women | 248 women | Sexual function at 6 months postpartum | High |
| Baytur et al. (2005) ^[44]^ | Turkey | Prospective cohort study | Women who were delivered vaginally or cesarean section and nulliparas attending our gynecology clinic with other symptoms | 68 women | Sexual function after childbirth | High |
| **Tool: Sexual Function Questionnaire (SFQ-28) ^[57]^** | | | | | |  |
| Song et al. (2014) ^[58]^ | Japan | Cross-sectional study | Mothers delivered a single baby at term | 435 women | Sexual function at 6 months after delivery | High |
| **Tool: Short form of the Pelvic Organ Prolapse/Urinary Incontinence Sexual Questionnaire (PISQ-12) ^[59]^** | | | | | |  |
| Dahlgren et al. (2022) ^[61]^ | Sweden | Prospective cohort study | All nulliparous women in early pregnancy registering for maternity health care in Region Örebro County | 958 women | Sexual function at early pregnancy, 8 weeks and 12 months postpartum | High |
| **Author (year)** | **Country** | **Study design** | **Study**  **Population** | **Sample Size** | **Outcome**  **(Definition)** | **Quality** |
| Gommesen et al. (2019) ^[60]^ | Denmark | Prospective cohort study | Primiparous women 12 months postpartum | 554 women | Sexual function at 12 months postpartum | High |
| **Tool: Sexual Health Outcomes in Women Questionnaire (SHOW-Q) ^[62]^** | | | | | |  |
| Yee et al. (2023) ^[63]^ | Canada | Prospective cohort study | Postpartum women | 160 women | Sexual activity at 8 to 10 weeks postpartum and at 6 to 8 months postpartum | High |
| **Tool: Shorter version of Female sexual function index (FSFI-6) ^[64]^** | | | | | |  |
| Bhat et al. (2022) ^[65]^ | India | Randomized Trial | Sexually active primiparous females in the postpartum period following normal vaginal delivery | 55 women | Sexual function, within 6 months postpartum | Low |
| **Tool: Sexual Function Questionnaire’s Medical Impact Scale (SFQ-MIS) ^[66]^** | | | | | |  |
| Levy et al. (2020) ^[68]^ | Israel | Cross-sectional study | Women aged 20 to 40 years and being 100 to 390 days postpartum | 382 women | Sexual Function, 100 to 390 days postpartum | High |
| Handelzalts et al. (2018) ^[67]^ | Israel | Survey | Primiparous and nulliparous women | 376 women | Sexual function, 100 to 390 days postpartum | High |

**Table S2.** Characteristics of included studies used the tools measuring sexual function/health in the postpartum period

| ID | **Author, Year** | **Representativeness of the exposed cohort** | **Selection of the non-exposed cohort** | **Ascertainment of exposure** | **Outcome not present at start of stud** | **Adjustment** | **Outcome assessment** | **follow-up long enough** | **Score** | **Quality** |
| --- | --- | --- | --- | --- | --- | --- | --- | --- | --- | --- |
| 1 | Yee et al., 2023 | 1 | 1 | 1 | 1 | 1 | 1 | 1 | 7 | High |
| 2 | Surucu et al, 2022 | **1** | **1** | **1** | 1 | 0 | 1 | 1 | **6** | High |
| 3 | Szollosi et al., 2022 | **1** | **1** | **1** | 1 | 1 | 1 | 1 | **7** | High |
| 4 | Dahlgren et al., 2022 | 1 | 1 | 1 | 1 | 0 | 1 | 1 | 6 | High |
| 5 | De Sousa et al., 2021 | **1** | **1** | **1** | 1 | 1 | 1 | 1 | **7** | High |
| 6 | Ugwu et al., 2020 | **1** | **1** | **1** | 1 | 0 | 1 | 1 | 6 | High |
| 7 | Spaich et al., 2020 | **1** | **1** | **1** | 1 | 0 | 1 | 1 | 6 | High |
| 8 | Spaich et al, 2020 | **1** | **1** | **1** | **1** | **0** | **1** | **1** | **6** | High |
| 9 | Gommesen et al., 2019 | 1 | 1 | 1 | 1 | 0 | 1 | 1 | 6 | High |
| 10 | Matthies et al., 2019 | **1** | **1** | **1** | 1 | 0 | 1 | 1 | 6 | High |
| 11 | Moghadam et al., 2019 | 1 | 1 | 1 | 1 | 0 | 1 | 1 | 6 | High |
| 12 | Kahramanoglu et al.,2017 | 1 | 1 | 1 | 1 | 0 | 1 | 1 | 6 | High |
| 13 | Yıldız et al., 2015 | 1 | 1 | 1 | 1 | 0 | 1 | 1 | 6 | High |
| 14 | Chang et al., 2015 | 1 | 1 | 1 | 1 | 0 | 1 | 1 | 6 | High |
| 15 | De Souza et al., 2015 | 1 | 1 | 1 | 1 | 0 | 1 | 1 | 6 | High |
| 16 | Lurie et al., 2013 | 1 | 1 | 1 | 1 | 0 | 1 | 1 | 6 | High |
| 17 | Citak et al., 2010 | 1 | 1 | 1 | 1 | 0 | 1 | 1 | 6 | High |
| 18 | Baksu et al., 2007 | 1 | 1 | 1 | 1 | 0 | 1 | 1 | 6 | High |
| 19 | Baytur et al., 2005 | 1 | 1 | 1 | 1 | 0 | 1 | 1 | 6 | High |

**Table S3.** Items for quality assessment of included studies with Cohort design

| ID | **Author, Year** | **Defined population** | **Given response rate** | **Described Statistical Analysis** | **justified sample size** | **Adjustment** | **Pretested/validated questionnaire** | **Outcome assessment** | **Score** | **Quality** |
| --- | --- | --- | --- | --- | --- | --- | --- | --- | --- | --- |
| 1 | Azarkish et al, 2022 | 1 | 1 | 1 | 1 | 0 | 1 | 1 | 6 | High |
| 2 | Bartels et al, 2021 | 1 | 1 | 1 | 0 | 0 | 1 | 1 | 5 | High |
| 3 | Zgliczynska et al, 2021 | 1 | 0 | 1 | 0 | 0 | 1 | 1 | 4 | High |
| 4 | Mirzaei et al, 2021 | 1 | 0 | 1 | 1 | 0 | 1 | 1 | 5 | High |
| 5 | Levy et al. 2020 | 1 | 0 | 1 | 0 | 0 | 1 | 1 | 4 | High |
| 6 | Szollosi et al., 2019 | 1 | 0 | 0 | 0 | 0 | 1 | 1 | 3 | Low |
| 7 | Szollosi et al., 2019 | 1 | 0 | 0 | 0 | 0 | 1 | 1 | 3 | Low |
| 8 | Handelzalts et al., 2018 | 1 | 1 | 1 | 0 | 0 | 1 | 1 | 5 | High |
| 9 | Banaei et al., 2018 | 1 | 1 | 0 | 1 | 0 | 1 | 1 | 5 | High |
| 10 | Rezaei et al., 2017 | 1 | 1 | 1 | 1 | 1 | 1 | 1 | 7 | High |
| 11 | Barbara et al., 2016 | 1 | 0 | 1 | 0 | 0 | 1 | 1 | 4 | High |
| 12 | Anbaran et al., 2015 | 1 | 0 | 0 | 1 | 0 | 1 | 1 | 4 | High |
| 13 | Dabiri et al., 2014 | 1 | 0 | 0 | 0 | 0 | 1 | 1 | 3 | Low |
| 14 | Song et al., 2014 | 1 | 1 | 1 | 0 | 0 | 1 | 1 | 5 | High |
| 15 | Baghdari et al., 2012 | 1 | 0 | 0 | 0 | 0 | 1 | 1 | 3 | Low |
| 16 | Hosseini et al., 2012 | 1 | 0 | 1 | 0 | 0 | 1 | 1 | 4 | High |
| 17 | Shirvani et al. ,2010 | 1 | 0 | 0 | 0 | 0 | 1 | 1 | 3 | Low |
| 18 | Klein et al. ,2009 | 1 | 1 | 1 | 1 | 0 | 1 | 1 | 6 | High |

**Table S4.** Items for quality assessment of included studies with Cross-sectional design

| ID | **Author, Year** | **Randomization (Yes/No)** | **Randomization (Method)** | **Blinding (Yes/No)** | **Blinding (Method)** | **An account of all patients** | **Total Score** | **Qualitative rating** |
| --- | --- | --- | --- | --- | --- | --- | --- | --- |
| 1 | Smith et al, 2022 | 1 | 1 | 1 | 0 | 1 | 4 | High |
| 2 | Bhat et al., 2022 | 1 | 0 | 0 | 0 | 1 | 2 | Low |
| 3 | Sheikhi et al. ,2020 | 1 | 0 | 0 | 0 | 1 | 2 | Low |

**Table S5.** Items for quality assessment of included studies with Trial design
